# Editorial: Language research on sustainability, ecology, and pro-environmental behavior

**DOI:** 10.3389/fpsyg.2023.1218961

**Published:** 2023-06-02

**Authors:** Jennifer Bruder, Salim Bouherar

**Affiliations:** ^1^Arts and Sciences, Carnegie Mellon University in Qatar, Doha, Qatar; ^2^Department of English Language and Literature, University of Sétif 2, Sétif, Algeria

**Keywords:** pro-environmental behavior, language, sustainability, ecolinguistics, story telling, climate justice, climate imagery

The consolidation of sustainable thinking and formative behavioral change necessitates that all environmental issues be communicated through an effective use of language. The way language is shaped to promote sustainable behaviors influences the feasibility of pro-environmental actions to increasingly prevail. Considering the power language must shape perceptions, attitudes, opinions and identity, means that researchers of these themes have much to offer to those interested in promoting pro-environmental behavior. The research presented in this Research Topic on language and environment is aimed diversely to highlight several rich avenues for future investigations.

Three papers in *Language research on sustainability, ecology, and pro-environmental behavior* center around the power of storytelling from unique lenses. In the first article, Huang et al. explore the impact of green brand stories on the sincerity and trust of customers. The language used in marketing is important for green products because consumers are susceptible to greenwashing. Greenwashing also causes distrust for green products and may result in consumers perceiving that green products lack authenticity. The study explores the effects of two variables (Customer Perceived Value, Need For Cognition) in green brand storytelling using Aristotle's Rhetorical Theory on the persuasion of customers. It concludes that green brand stories which appeal to consumers using humor, empathy, and metaphor along with providing elements promoting credibility (i.e., concrete numbers, green business certifications and clear contact details) build trust, also shows that logic was less helpful for promoting brand sincerity.

In the second paper, Doehring et al. examine the impact of knowledge sharing through storytelling on improving restoration of freshwater ecosystems. The authors interview five catchment groups to unveil their water restoration stories as a source of inspiration and knowledge sharing. The findings of the study suggest that storytellers provide insights and reflections for both contemporary needs and future responsibilities of the next generations, while conveying the portrayal of struggle and threats facing their ambitions. The use of storytelling can serve as a powerful tool for sustainability initiatives and the methods and results of this study can be extrapolated to other types of sustainability research.

Third, van Kempen et al. showcase the value of teaching storytelling skills for environmental science communication amongst university students who are typically taught to disseminate science using only formal techniques. In their study, the authors convey their experiences in teaching university students how to write impactful environment-focused stories for general audiences in cross-cultural educational settings. Indeed, one issue with scientific writing is its inaccessibility to general audiences. In the case of the environment, there is, arguably, a great need to teach students communication skills that can improve their ability to educate and connect with lay people. The authors of this paper asked students to write blogs on topics of their interest and found evidence for change in student perspectives on environment related issues. They also highlight the need for more intercultural collaboration and learning opportunities on the topics of technology and environmental issues.

In line with the significance of understanding student cognition around sustainability, Huang examined 501 collaborative essays written by more than 2,000 Chinese students on sustainability to investigate student perceptions about sustainability as well as language demonstrating beliefs about who are the responsible actors. Huang identified three main themes in the essays: environmental, economic, and social issues. In addition to that, evidence for superficial green talk and anthropomorphism in the student discourse was also found. Further, although students demonstrated a high level of knowledge about environment and sustainability, they tended to express views that government and business sectors should act as the main change agents, thus detaching themselves from being active participants in sustainable development.

Two papers in *Language research on sustainability, ecology, and pro-environmental behavior* explore different perspectives on social and environmental injustices and how language can be used to mitigate inequalities and related environmental crises. In the first paper, Marsili et al. write about the positive effects of promoting Environmental Health Literacy (EHL) amongst people living in contaminated areas in Italy. Contaminated communities are characterized by vulnerable people living in socio-economic deprivation. These communities often live on the edge of environmentally hazardous industry, which also impacts their health with generally low EHL. Establishing stronger communication abilities empowers these communities to not only advocate for improved environmental outcomes, but also to reduce social vulnerabilities and future health inequalities through improved awareness.

Fine discusses the sociolinguistic injustices related to efforts to fight climate issues in the United States. She examines how social injustice can impede climate movement activists' voices and neglect or marginalize their struggle. The empirical results of the study suggest that social injustice prevents activists from taking part in the climate manifestations. The paper concludes that aspects manifesting sociolinguistic injustice are restrained in the following: the use of a monolingual communication presented in the English language, restricting the variants of English to the American English only, and the use of language stereotypes.

Finally, Jones et al. provide a unique perspective on this Research Topic's focus by highlighting the combined role of visual imagery and language, through the power of online tools, to spread sustainable messaging, including both pro and contra environmental views. Through three case studies on iconic images, the authors analyze how the images have been utilized by various groups to create memes which demonstrated their ability to shape identity, promote thought and encourage activism. The online circulation of memes highlights the power of social media to promote ideologies both for and against environmental activism and shed light on how critical language combined with images shape identity, thought and opinions in environmental communication.

We hope that the reader will find useful insights highlighted in this Research Topic for their own research and gain further appreciation for the power of language in shaping themes related to sustainability, ecology, and pro-environmental behaviors.

## Author contributions

JB and SB contributed equally to the writing and conceptualization of this article. All authors contributed to the article and approved the submitted version.

